# Left ventricular myocardial strain assessed by cardiac magnetic resonance feature tracking in patients with rheumatoid arthritis

**DOI:** 10.1186/s13244-020-00948-6

**Published:** 2021-01-07

**Authors:** Wojciech Tański, Paweł Gać, Angelika Chachaj, Grzegorz Mazur, Rafał Poręba, Andrzej Szuba

**Affiliations:** 1 Department of Internal Medicine, 4Th Military Hospital, Weigla 5, 50-981 Wroclaw, Poland; 2 Centre for Diagnostic Imaging, 4Th Military Hospital, Weigla 5, 50-981 Wroclaw, Poland; 3 Department of Hygiene, Wroclaw Medical University, Mikulicza-Radeckiego 7, 50-368 Wrocław, Poland; 4 Department of Angiology, Hypertension and Diabetology, Wroclaw Medical University, Borowska 213, 50-556 Wrocław, Poland; 5 Department of Internal and Occupational Diseases, Hypertension and Clinical Oncology, Wroclaw Medical University, Borowska 213, 50-556 Wrocław, Poland

**Keywords:** Cardiac magnetic resonance, Left ventricular myocardial strain, Rheumatoid arthritis

## Abstract

**Purpose:**

The aim of the study was to assess a relationship between the occurrence of rheumatoid arthritis (RA) and its selected clinical parameters, and left ventricular myocardial strain.

**Material and methods:**

Fifty-six subjects were qualified for the study: 30 RA patients and 26 subjects without rheumatoid diseases. The study design included taking medical history, assessment of the disease activity using selected scales of activity, collecting samples of venous blood to assess selected laboratory parameters and the assessment of cardiac magnetic resonance (CMR). Using the feature tracking method, the following parameters of the left ventricular myocardial strain were assessed: longitudinal strain (LS), radial strain (RS) and circumferential strain (CS).

**Results:**

Regarding global values, peak LS and peak CS were statistically significantly lower in RA patients than in the control group. In the whole study group, the factors independently related to low global LS peaks were as follows: occurrence of RA, occurrence of arterial hypertension, increased activity of antibodies against cyclic citrullinated peptide and increased concentration of neutrophil gelatinase-associated lipocalin. The occurrence of RA, occurrence of diabetes, tobacco smoking, higher activity of antibodies against cyclic citrullinated peptide and current use of methotrexate are the risk factors for low peak of global CS. The current use of steroids constitutes a protecting factor against low global CS peaks.

**Conclusion:**

In subjects with no clinically manifested cardiac damage, RA is associated with a deteriorated left ventricular systolic function assessed by left ventricular myocardial strain measured by CMR feature tracking.

## Key points

- Left ventricular systolic dysfunction assessed by myocardium strain on MRI is observed in rheumatoid arthritis.
- Left ventricular systolic dysfunction in rheumatoid arthritis manifests both general and regional strains.
- Rheumatoid arthritis is an independent risk factor for reduced peak global longitudinal and circumferential strain.

## Introduction

Rheumatoid arthritis (RA) belongs to the most frequently diagnosed rheumatic diseases. Its prevalence in the population reaches 0.5% [1]. As shown by recent studies, RA patients belong to a group characterised by increased cardiovascular risk [2, 3]. In the course of rheumatoid arthritis, one observes changes in the heart morphology and function, shown primarily by echocardiography [4]. Studies on the relationship between RA and structural and functional cardiac changes using magnetic resonance imaging are less numerous. Ntusi et al. showed that RA predisposes to higher incidence of left ventricular enlargement, deterioration of the left ventricular systolic function, myocardial swelling, myocardial fibrosis and increased myocardial extracellular space volume [5]. Kobayashi et al. estimated that in patients with RA the incidence of fibrosis foci in the left ventricular myocardium was 32–39%, incidence of active inflammation in the left ventricular myocardium—12%, and incidence of perfusion deficits in the left ventricular myocardium—11% [6, 7]. Also, Giles et. al. documented that RA was associated with a reduced left ventricular ejection fraction and reduced left ventricular mass [8].

The basic function of the heart is its systolic function. A standard method of assessing the systolic function of the heart is, regarding its global function—quantitative assessment of the left ventricular systolic fraction, and regarding its regional function—qualitative, visual assessment of contractility of particular myocardial segments. Over the last few years, considering limitations of standard methods to assess left ventricular systolic function, a more precise method of assessment has been proposed, namely quantitative analysis of left ventricular myocardial strain [9, 10]. Initially, methods of echocardiographic assessment of left ventricular myocardial strain were developed [11]. Left ventricular myocardial strain assessed by magnetic resonance feature tracking is a qualitative method of assessing myocardial contraction characterised by high repeatability and relatively low dependence on external factors [12]. Strain analysis involves the assessment of three basic directions of myocardial strain during its contraction—longitudinal shortening, circumferential shortening and radial thickening [13].

So far, literature has reported the assessment of left ventricular (LV) systolic function disorders with the use of left ventricular myocardial strain in certain diseases of interest for rheumatology, i.e. Duchenne muscular dystrophy, amyloidosis or systemic sclerosis [14–16]. According to the authors, it seems reasonable to verify a hypothesis assuming the existence of the same relationship in patients with a much more common disease in the society, i.e. rheumatoid arthritis.

The aim of the study was to assess a relationship between the occurrence of rheumatoid arthritis and its selected clinical parameters, and left ventricular myocardial strain assessed with the use of cardiac magnetic resonance feature tracking.

## Material and methods

**Fig. 1:**
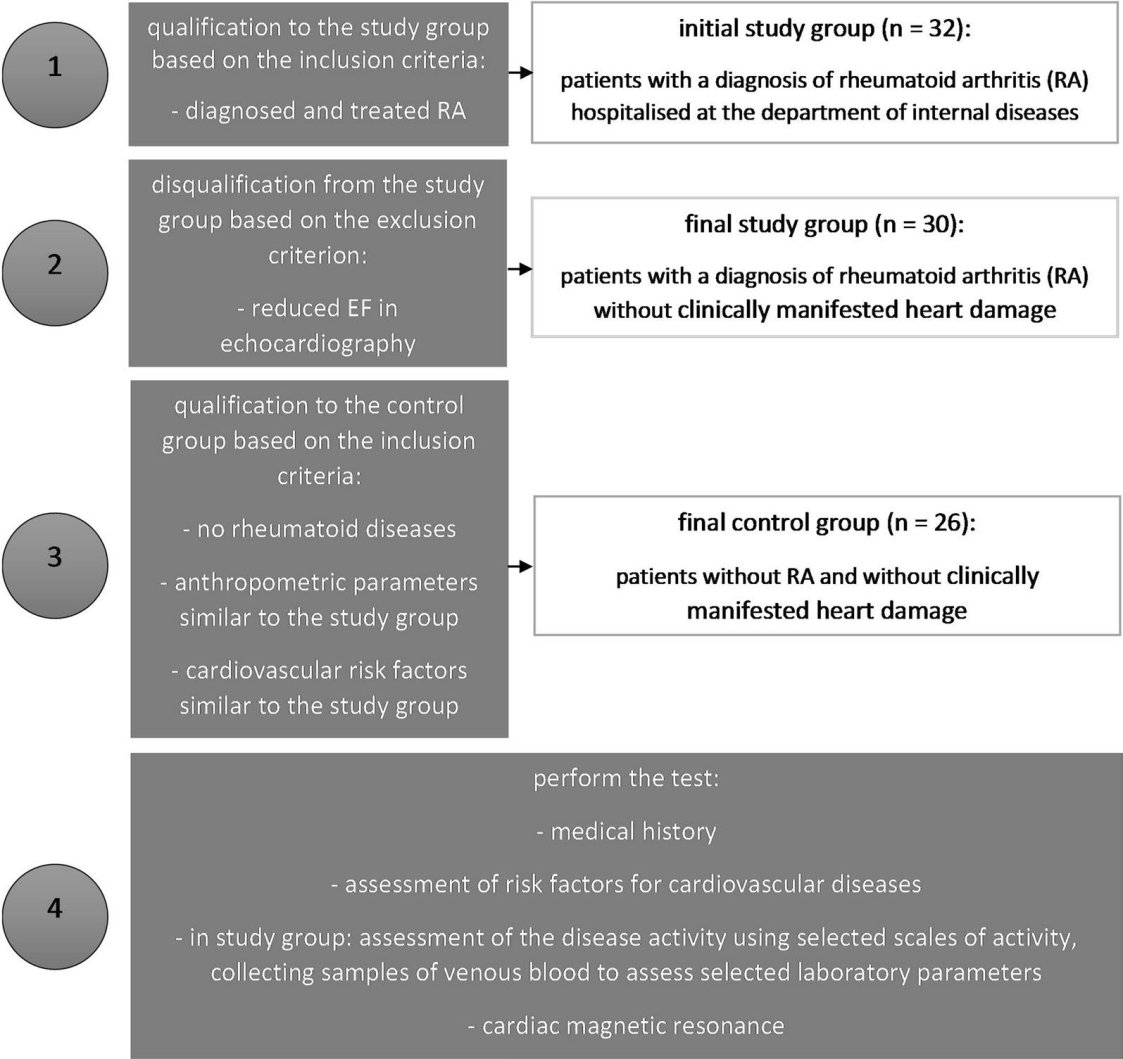
The complete study protocol

In total, 56 subjects were qualified for this prospective study. During the first stage, consecutive patients with a diagnosis of rheumatoid arthritis (RA) hospitalised at the department of internal diseases of a single site were enrolled in the study group. While qualifying RA patients, subjects with clinically manifested heart damage (reduced ejection fraction in routine echocardiography) were excluded. At this stage of the study, 30 RA patients (20 females and 10 males) aged 49.93 ± 13.68, with a mean body mass index (BMI) of 25.41 ± 4.09 kg/m^2^, were enrolled in the study. Then, subjects with no rheumatoid diseases, having similar anthropometric features and similar characteristics regarding risk factors for cardiovascular diseases were added to the analysed group. Twenty-six subjects added in this way (16 females and 10 males) aged 49.42 ± 7.16, with BMI of 25.51 ± 3.01 kg/m^2^, formed a control group. General characteristics of the study groups are presented in Table 1. Selected clinical and laboratory features of patients with rheumatoid arthritis are presented in Table 2. The complete study protocol is presented in Fig. 1.

**Table 1:** General characteristics of the study group of patients

	RA	CON
Number [*n*/%]	30/100.0	26/100.0
Gender [*n*/%]		
Men	10/33.3	10/38,5
Women	20/66.7	16/61.5
Age [years]	49.83 ± 13.68	49.42 ± 7.16
Height [m]	1.65 ± 0.08	1.68 ± 0.07
Weight [kg]	70.67 ± 13.67	71.75 ± 9.01
Body mass index (BMI) [kg/m^2^]	25.41 ± 4.09	25.51 ± 3.01
Body surface area (BSA) [m^2^]	1.76 ± 0.17	1.81 ± 0.13
Overweight/obesity [*n*/%]		
Normal body mass	14/46.7	12/46.1
Overweight/obesity	16/53.3	14/54.9
Coexistence of cardiovascular risk factors [*n*/%]		
Arterial hypertension	8/26.7	5/19.2
Type 2 diabetes	3/10.0	2/7.6
Smoking	2/6.7	3/11.5

No statistical differences between the studied groups

**Table 2:** Selected clinical parameters in the group of patients with rheumatoid arthritis

Duration of illness [years]	8.35 ± 6.56
C-reactive protein (CRP) [mg/l]	17.47 ± 17.23
Rheumatoid factor (RF) [IU/ml]	163.83 ± 329.98
Anti-cyclic citrullinated peptide (anti-CCP) [EU/ml]	333.69 ± 479.44
Seropositivity [*n*/%]	19 / 63.3
Disease activity index DAS28	6.48 ± 0.52
Collective assessment of health by the patient and the doctor (VAS)	82.50 ± 4.08
Neutrophil gelatinase-associated lipocalin (NGAL) [ng/ml]	12.79 ± 8.80
Steroids [*n*/%]	20/66.7
Methotrexate [*n*/%]	22/73.3
Other disease-modifying drugs [*n*/%]	12/40.0
Salazopyrin	1/3.3
Leflunomide	7/23.3
Cyclosporine	3/10.0
Chloroquine	1/3.3

The project received the approval of the local ethics committee. The studies were performed in accordance with the principles of Good Clinical Practise. The study participants give their written, informed consent to take part in the research project. The study design included taking medical history, assessment of the disease activity using selected scales of activity, collecting samples of venous blood to assess selected laboratory parameters and the assessment of cardiac magnetic resonance (CMR).

Based on the medical history taken, data on the principal disease and comorbidities were obtained, and risk factors for cardiovascular system diseases were determined. The assessment of disease activity was based on the scales: DAS28 index and VAS cumulative assessment of the health state by patient and physician.

Using the collected samples of venous blood, selected laboratory parameters characterising the course of rheumatoid arthritis were determined: concentration of C-reactive protein (CRP), activity of rheumatoid factor (RF), concentration of neutrophil gelatinase-associated lipocalin (NGAL) and activity of antibodies against cyclic citrullinated peptide (anti-CCP); also, seropositivity was assessed. The determinations were based on tests used in the department of laboratory diagnostics providing services for the department of internal diseases, where the study subjects were hospitalised. The department of diagnostic imaging is certified as part of the Randox International Quality Assessment Scheme (RIQAS).

Cardiac magnetic resonance was performed with the use of a single 1.5-T device Magnetom Aera (Siemens Healthcare, Forchheim, Germany)—according to the same protocol in all subjects. The image acquisition was gated by ECG, and it was performed during the patient's breath-hold. For the purposes of the present study, CINE-balanced SSFP (steady-state free precession sequence) sequences were analysed. The following sequence technical parameters were used: repetition time/echo time (ms), 45.60/1.19; flip angle, 70°; field of view, 350 mm^2^; matrix, 256 × 154; slice thickness, 10 mm with a 2-mm gap; mean temporal resolution of 30 ms with 30 phases per cardiac cycle. CINE sequences were typically registered in the long axing in 2-chamber, 3-chamber and 4-chamber views and in the short axis of the left ventricle. Medis Suite MR software was used to assess post-processing CMR images (Medis, Leiden, The Netherlands).

In the first stage of the CMR image analysis, using the QMass application (Medis, Leiden, The Netherlands), cardiac chamber size and functional parameters of the left ventricle were assessed.

**Fig. 2:**
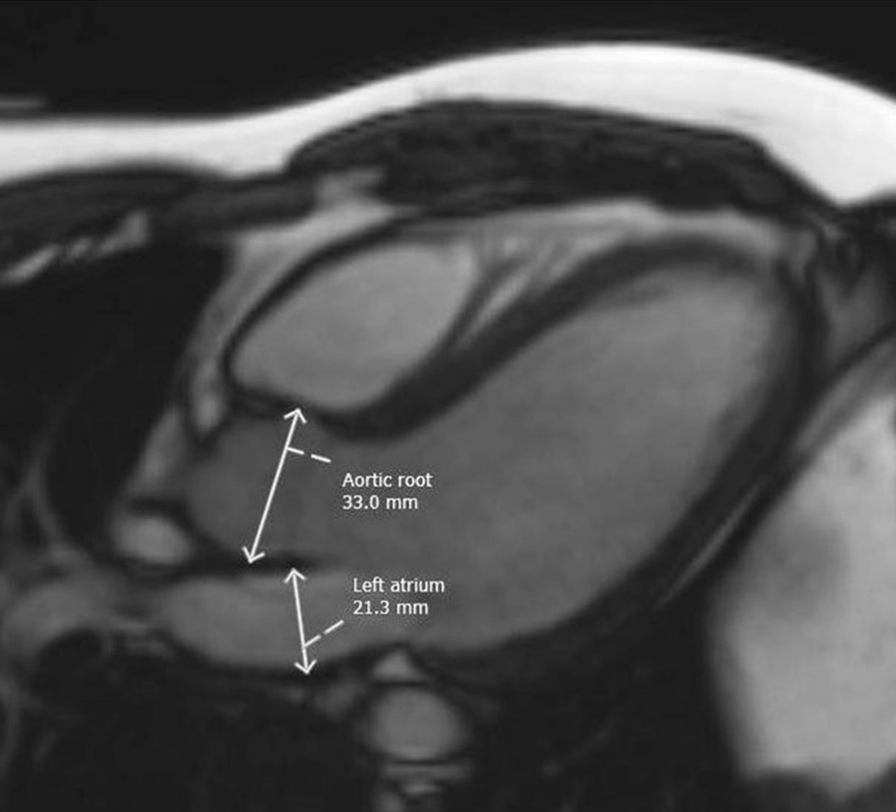
The method of measuring LA and Ao in a 3-chamber CMR projection

**Fig. 3:**
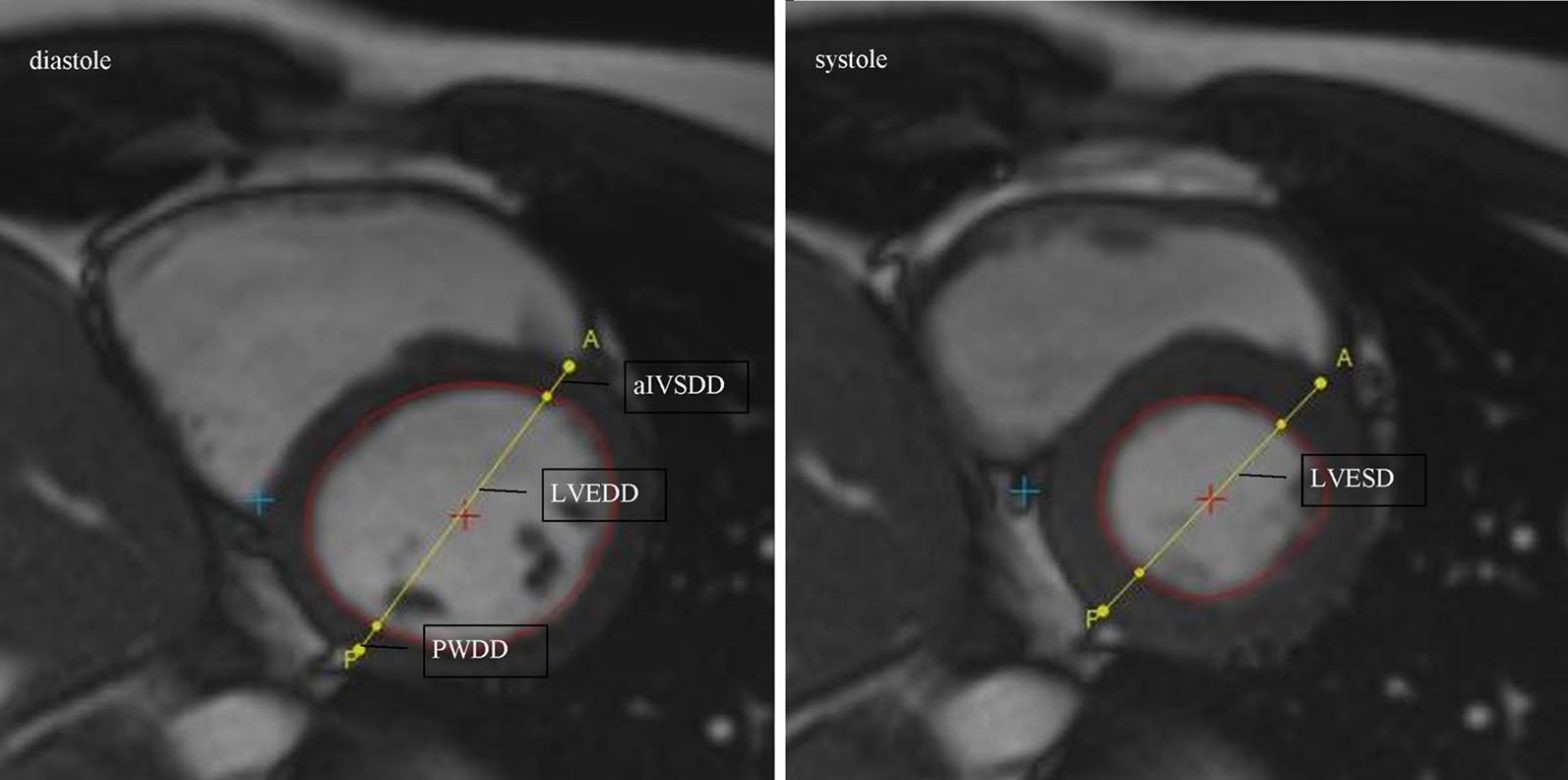
The method of measuring aIVSDD, PWDD, LVEDD and LVESD in a short-axis CMR projection

In the 3-chamber view along the long axis, the size of the left atrium (LA) and size of aortic root (Ao) were assessed. LA and Ao measurements were performed manually, as shown in Fig. 2. In a short axis view, at the level of basal-mid segments, the measurements included anterior interventricular septum diastolic dimension (aIVSDD), posterior wall diastolic dimension (PWDD), left ventricular end diastolic dimension (LVEDD) and left ventricular end-systolic dimension (LVESD). The aIVSDD, PWDD, LVEDD and LVESD measurements were performed semi-automatically with the left ventricular diameter wizard, as shown in Fig. 3.

The 2-chamber view, 4-chamber view in the long axis and the LV view in the short axis were used for volumetric assessment of the left ventricular function. End-diastolic volume (EDV), end-systolic volume (ESV), stroke volume (SV) and ejection fraction (EF) were calculated. The left ventricular functional parameters were indexed to body surface area (BSA). In addition, left ventricular mass (LVM) and left ventricular mass index (LVMI) were assessed. Left ventricular volumetry analysis was performed semi-automatically with the ventricular analysis wizard.

In a subsequent stage of the CMR image analysis, left ventricular myocardial strain was assessed with the use of the QStrain RE application (Medis, Leiden, The Netherlands). The short-axis and long-axis images were imported to the application. Then, end-systolic and end-diastolic images were selected, and epicardial and endocardial contours were manually drawn. Using the feature tracking method, the application automatically assessed the parameters of the left ventricular myocardial strain: longitudinal strain (LS), radial strain (RS) and circumferential strain (CS). Peak strain values were assessed for each of 16 AHA segments of the left ventricle: 6 basal segments, 6 mid-cavity segments and 4 apical segments [17]. In the basal and mid-cavity sections, the anterior, anteroseptal, inferoseptal, inferior, inferolateral and anterolateral segments were analysed. By contrast, in the apical section, the anterior, septal, inferior and lateral segments were analysed. Moreover, peak strain was assessed for the whole basal, mid-cavity and apical layers. Also, global longitudinal strain (GLS), global radial strain (GRS) and global circumferential strain (GCS) were measured.

All CMR examinations were assessed by the same radiologist with over 5 years of experience in assessing this type of examination (about 150 examinations per year), certified by the Polish Medical Imaging Society in the field of imaging diagnostics of the heart and vessels.

Statistical analyses were performed with the use of “Dell Statistica 13″ statistical package (Dell Inc., USA). Quantitative variables are presented as arithmetic means ± standard deviations. The distribution of variables was verified by the Shapiro–Wilk test. In the case of independent quantitative variables with normal distribution, further statistical analysis was based on the one-tailed parametric *t*-test. For variables showing distribution other than normal, nonparametric Mann–Whitney U test was used for independent quantitative variables. Results for qualitative variables were presented as percentage sizes/values. Chi-square test was used in the further analysis for independent qualitative variables. To assess a relationship between the study parameters, a stepwise backward multivariable regression analysis was performed. The parameters of the model obtained in the regression analysis were estimated using the least squares method. Values at *p* < 0.05 were considered statistically significant.

## Results

The study groups did not differ in the basic anthropometric parameters or in coexistence of the study risk factors for cardiovascular diseases. As mentioned above, basic clinical features of the study groups are presented in Table 1.

The study group of RA patients was characterised by a mean time of principal disease of 8.35 ± 6.56 years, mean activity of rheumatoid factor of 163.83 ± 329.98 IU/mL, and seropositivity observed in 63.3% of the study patients. A vast majority of the study RA patients were treated with methotrexate (73.3%) and steroids (66.7%) during the study. At the same time, 40% patients also received other drugs modifying the course of the disease, especially leflunomide (23.3%). Selected clinical parameters assessed in the study group of patients with rheumatoid arthritis are presented in Table 2.

A comparative analysis did not show statistically significant differences between the RA patients and the control group in the basic parameters of CINE-balanced SSFP cardiac magnetic resonance. The study subjects with RA subjects from the control group did not differ either in the left ventricular dimensions, left ventricular mass, or volumetric parameters of the left ventricular systole. Basic parameters of CINE-balanced SSFP cardiac magnetic resonance imaging in the study groups are presented in Table 3.

**Table 3:** Basic parameters of CINE-balanced SSFP cardiac magnetic resonance imaging in the study groups

	RA	CON
*Heart cavity dimensions*		
Left atrium diameter (LA) [mm]	33.23 ± 5.07	33.66 ± 3.85
Aortic root diameter (Ao) [mm]	29.83 ± 3.83	32.39 ± 3.04
Left ventricular end-diastolic diameter (LVEDD) [mm]	55.74 ± 30.85	56.98 ± 7.81
Left ventricular end-systolic diameter (LVESD) [mm]	33.02 ± 11.92	37.66 ± 9.82
Anterior intra-ventricular septum diastolic Diameter (aIVSDD) [mm]	9.28 ± 1.92	8.58 ± 1.43
Posterior wall diastolic diameter (PWDD) [mm]	8.55 ± 1.52	7.28 ± 1.19
Left ventricular mass (LVM) [g]	105.59 ± 23.86	114.59 ± 27.57
Left ventricular mass index (LVMI) [g/m^2^]	59.91 ± 11.80	60.12 ± 13.78
*Left ventricular systolic function*		
End-diastolic volume index (EDVi) [ml/m^2^]	81.08 ± 19.24	84.24 ± 18.31
End-systolic volume index (ESVi) [ml/m^2^]	31.57 ± 12.17	33.55 ± 11.36
Stroke volume index (SVi) [ml/m^2^]	49.51 ± 11.44	57.29 ± 13.02
Ejection fraction (EF) [%]	63.62 ± 7.39	63.46 ± 5.28

No statistical differences between the studied groups

**Fig. 4:**
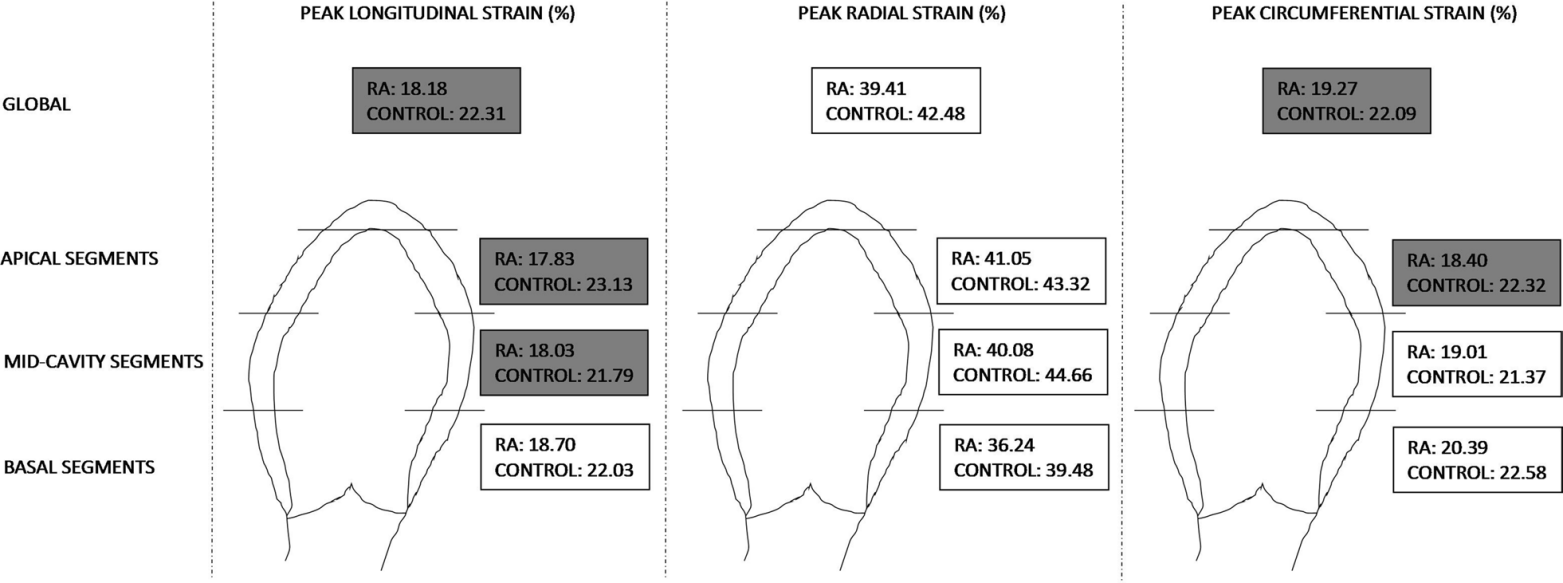
Parameters of left ventricular myocardial strain assessed by cardiac magnetic resonance feature tracking in the study groups of patients

The comparative analysis revealed, however, a presence of statistically significant differences in the left ventricular myocardial strain parameters assessed by cardiac magnetic resonance feature tracking between the RA patients and the control group. Regarding global values, peak GLS and peak GCS were statistically significantly lower in RA patients than in the control group patients. A complete analysis of particular LV layer showed that for the apical layer, peak LS and peak CS were significantly lower in RA patients versus patients from the control group. For the mid-cavity layer, there was a statistically significant difference regarding LS, whose peak was significantly lower in the RA group as compared with control. And for the basal layer, no statistically significance differences in myocardial strain were revealed. Parameters of left ventricular myocardial strain assessed by cardiac magnetic resonance feature tracking in the study groups of patients are presented in Fig. 4.

**Fig. 5:**
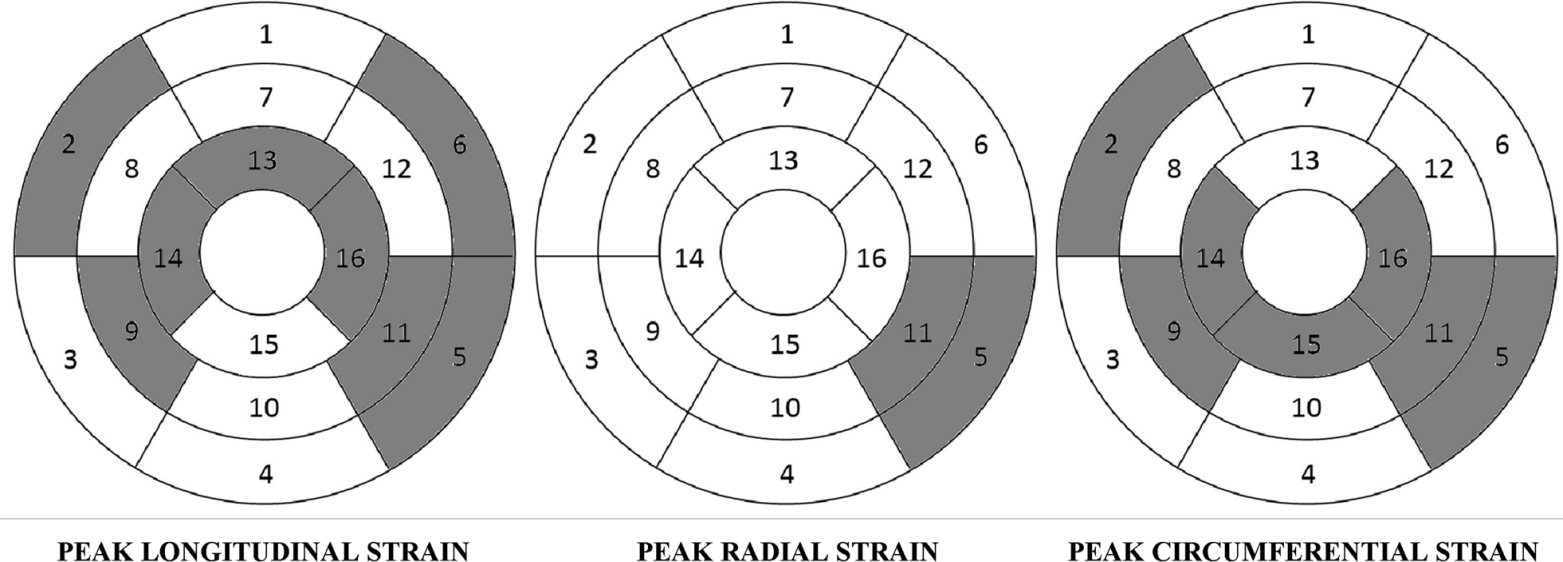
Left ventricular myocardial segments with statistically significantly lower peak of strain values in the group of patients with rheumatoid arthritis compared to the control group

The analysis of particular 16 segments of the left ventricle showed numerous statistically significant differences between the groups regarding myocardial strain. Peak of longitudinal strain in RA patients compared to patients from the control group was statistically significantly lower in segments 2, 5, 6, 9, 11, 13, 14 and 16. A significantly lower peak of circumferential strain in RA patients compared to patients from the control group was observed for segments 2, 5, 9, 11, 14, 15 and 16. And the peak of radial strain in RA patients in comparison with patients from the control group was significantly lower in segments 5 and 11. Complete results of the analysis of left ventricular strain assessed by CMR feature tracking in the study groups are presented in Table 4 and Fig. 5.

**Table 4:** Parameters of left ventricular myocardial strain assessed by cardiac magnetic resonance feature tracking in the study groups of patients

Segments	Longitudinal strain [%]		Radial strain [%]		Circumferential strain [%]	
	RA	CON	RA	CON	RA	CON
*Segmental strain: basal segments*						
1: Anterior	−20.28 ± 4.47	−22.58 ± 4.18	40.98 ± 20.89	42.15 ± 15.57	−21.45 ± 4.15	−21.34 ± 5.26
2: Anteroseptal	−18.75 ± 5.19 *	−22.67 ± 7.18	38.48 ± 20.48	38.18 ± 18.48	−19.78 ± 4.56 *	−24.02 ± 3.57
3: Inferoseptal	−19.87 ± 5.64	−19.82 ± 3.49	30.13 ± 17.64	35.46 ± 22.06	−22.16 ± 3.98	−23.04 ± 4.96
4: Inferior	−21.27 ± 6.21	−22.01 ± 5.19	35.15 ± 15.65	38.16 ± 17.89	−20.86 ± 4.87	−21.84 ± 3.86
5: Inferolateral	−17.45 ± 6.11 *	−23.02 ± 4.48	33.56 ± 14.18 *	40.78 ± 12.08	−19.58 ± 3.87 *	−23.26 ± 3.23
6: Anterolateral	−14.56 ± 8.01 *	−22.09 ± 5.95	39.15 ± 22.01	42.16 ± 19.48	−18.52 ± 6.02	−22.01 ± 6.21
Whole layer	−18.70 ± 6.54	−22.03 ± 5.49	36.24 ± 17.06	39.48 ± 18,86	−20.39 ± 4.86	−22.58 ± 4.59
*Segmental strain: mid-cavity segments*						
7: Anterior	−21.15 ± 5.48	−23.58 ± 6.19	43.08 ± 13.86	44.06 ± 16.84	−22.02 ± 4.89	−21.98 ± 6.24
8: Anteroseptal	−17.97 ± 4.18	−20.02 ± 4.11	44.11 ± 20.36	45.89 ± 17.69	−19.57 ± 3.59	−19.02 ± 3.54
9: Inferoseptal	−15.86 ± 8.18 *	−23.76 ± 8.42	39.45 ± 26.02	42.13 ± 23.65	−16.03 ± 4.12 *	−22.94 ± 4.87
10: Inferior	−18.96 ± 7.16	−19.52 ± 6.86	37.18 ± 19.18	40.63 ± 25.17	−17.58 ± 5.63	−18.62 ± 4.98
11: Inferolateral	−17.02 ± 4.26 *	−24.85 ± 4.29	36.84 ± 15.98 *	45.15 ± 12.68	−18.65 ± 4.02 *	−24.63 ± 3.47
12: Anterolateral	−17.23 ± 3.45	−19.03 ± 6.25	45.16 ± 17.98	50.11 ± 21.06	−20.21 ± 5.12	−21.03 ± 4.26
Whole layer	−18.03 ± 5.96 *	−21.79 ± 5.15	40.08 ± 18.96	44.66 ± 16.87	19.01 ± 4.67	21.37 ± 4.56
*Segmental strain: apical segments*						
13: Anterior	−19.02 ± 5.12 *	−22.85 ± 5.65	49.11 ± 17.09	48.18 ± 20.03	−18.63 ± 4.58	−18.60 ± 3.59
14: Septal	−19.11 ± 3.48 *	−25.19 ± 3.94	35.16 ± 18.35	40.18 ± 19.56	−20.45 ± 4.69 *	−24.64 ± 3.99
15: Inferior	−18.25 ± 6.11	−22.02 ± 6.23	34.58 ± 16.60	39.65 ± 15.37	−17.69 ± 3.69 *	−24.01 ± 4.19
16: Lateral	−14.97 ± 5.18 *	−22.49 ± 4.87	45.36 ± 17.33	45.29 ± 16.66	−16.84 ± 4.60 *	−22.07 ± 3.78
Whole layer	−17.83 ± 5.91 *	−23.13 ± 4.97	41.05 ± 17.88	43.32 ± 18.06	−18.40 ± 4.18 *	−22.32 ± 3.89
*Global strain*						
All segments	−18.18 ± 5.01 *	−22.31 ± 4.85	39.41 ± 18.16	42.48 ± 17.96	−19.27 ± 4.52*	−22.09 ± 4.34

With the use of stepwise backward multivariable regression analysis, a relationship between anthropometric, clinical and laboratory parameters, RA occurrence and coexistence of selected risk factors for cardiovascular diseases versus global longitudinal strain, global radial strain and global circumferential strain were assessed. The following statistically significant models of regression are achieved:

$${\text{GLS }} = \, - {4}0.{663 } + { 3}.{\text{519 RA }} + { 2}.{\text{939 arterial hypertension }} + \, 0.00{\text{5 anti}} - {\text{CCP }} + \, 0.{\text{333 NGAL}};$$

$${\text{GCS }} = \, - { 37}.0{42 } + { 2}.{\text{332 RA }} + { 8}.{3}0{\text{9 diabetes }} + { 4}.{\text{651 tobacco smoking }} + \, 0.00{\text{4 anti}} - {\text{CCP }}{-}{ 3}.{\text{175 steroids }} + { 4}.0{\text{57 methotrexate}}.$$

On the basis of the obtained models, it was documented that in the study group of patients, the factors independently related to low GLS peaks were as follows: occurrence of RA, occurrence of arterial hypertension, increased activity of antibodies against cyclic citrullinated peptide and increased concentration of neutrophil gelatinase-associated lipocalin (Table 5A). In the same group, the occurrence of RA, occurrence of diabetes, tobacco smoking, higher activity of antibodies against cyclic citrullinated peptide and current use of methotrexate are the risk factors for low-peak GCS. At the same time, the current use of steroids constitutes a protecting factor against low GCS peaks (Table 5B). Complete results of the regression analysis in the study group of patients are presented in Table 5.

**Table 5:** Results of regression analysis in the study group of patients

Model for: global longitudinal strain (GLS) [%]					
	Intercept	RA ^#^	Arterial hypertension ^#^	Anti-cyclic citrullinated peptide (anti-CCP) [EU/ml]	Neutrophil gelatinase-associated lipocalin (NGAL) [ng/ml]
*A. Risk factors for global longitudinal strain (GLS) reduction*					
Regression coefficient	−40.663	3.519	2.939	0.005	0.333
SEM of Rc	15.441	1.241	1.302	0.002	0.121
p	0.005	0.006	0.042	0.027	0.009

Model for: global circumferential strain (GCS) [%]							
	Intercept	RA^#^	Type 2 diabetes^#^	Smoking^#^	Anti-cyclic citrullinated peptide (anti-CCP) [EU/ml]	Steroids ^#^	Methotrexate^#^
*B. Risk factors for global circumferential strain (GCS) reduction*							
Regression coefficient	−37.042	2.332	8.309	4.651	0.004	−3.175	4.057
SEM of Rc	5.279	0.994	2.336	1.916	0.002	1.546	1.592
p	0.001	0.023	0.001	0.019	0.035	0.045	0.014

^#^Dichotomous variable, where 1- no, 2- yes

## Discussion

On the basis of the presented results, it may be concluded that in subjects with no clinically manifested cardiac damage, rheumatoid arthritis is associated with a deteriorated left ventricular systolic function assessed by left ventricular myocardial strain measured by cardiac magnetic resonance feature tracking. Although a comparative analysis showed no statistically significant differences regarding basic parameters of CINE-balanced SSFP cardiac magnetic resonance imaging, in this aspect of left ventricular ejection fraction between RA group and control, there were numerous statistical differences regarding parameters of left ventricular myocardial strain assessed by cardiac magnetic resonance feature tracking between the groups of patients. The observed statistically significantly lower-peak strain values in the RA group vs control are interpreted as worse myocardium contractility in this group of patients. It was also documented that deteriorated left ventricular systolic function in RA patients refers to both global and regional strain parameters. Lower global contractility in RA patients is related to longitudinal and circumferential shortening. It has been shown that peak GLS and peak GCS were statistically significantly lower in RA patients than in the control group patients. On the other hand, lower regional contractility in RA patients affects a number of specific segments, especially associated with the interventricular septum and left ventricular lateral wall. It has been documented that in RA patients versus patients in the control group, peak of longitudinal strain was statistically significantly lower in segments 2, 5, 6, 9, 11, 13, 14 and 16; peak of circumferential strain was statistically significantly lower in segments 2, 5, 9, 11, 14, 15 and 16; and peak of radial strain was statistically significantly lower in segments 5 and 11. All the above-mentioned myocardial segments, except for segments 13 (anterior apical) and 15 (inferior apical), are located within the interventricular septum and left ventricular lateral wall. Moreover, it has been demonstrated that the occurrence of rheumatoid arthritis in the study group is a risk factor for deteriorated left ventricular systolic function, independent of other variables, presenting as lower-peak global longitudinal strain and peak global circumferential strain. The stepwise backward multivariable regression analysis showed that rheumatoid arthritis was statistically significantly correlated with GLS and GCS values, independently of other anthropometric, clinical and laboratory variables considered in the achieved models.

A relationship between rheumatoid arthritis and left ventricular systolic function assessed by strain in cardiac magnetic resonance has been discussed in the current literature only regarding a few studies. In the studies of above-quoted Ntusi et al., a case-to-case comparison of 39 RA patients with 39 patients from the control group showed that peak systolic circumferential strain and peak diastolic circumferential strain rate were diminished in RA patients, and the left ventricular ejection fraction did not differentiate the study groups [5]. In the studies of Bissell et al., 76 RA patients without cardiovascular comorbidities and diabetes were compared with 26 healthy subjects. The comparison revealed that RA was correlated with reduced left ventricular ejection fraction, reduced absolute values of mid-systolic strain rate and lower late/active diastolic strain rate, and that this relationship was independent of age, sex and classic risk factors for cardiovascular system diseases [18]. In the studies of Ntusi NAB et al., 4 study groups were compared: 23 subjects with RA and without cardiovascular comorbidities, 46 subjects with RA and with cardiovascular comorbidities, 13 subjects without RA with a cardiovascular disease (CVD) and 50 healthy subjects in the control group. The analysed groups did not differ in the left ventricular ejection fraction, but disorders in the left ventricular systolic function manifested as deterioration of mid-short-axis circumferential systolic strain and peak diastolic strain rate were increasing in the groups in the following order: healthy subjects < subjects with RA without CVD = subjects with CVD without RA < subjects with RA and CVD [19]. This relationship is also indirectly indicated in another study of Ntusi et al., which compared changes in the heart morphology and function in a group of 32 subjects with rheumatoid diseases (including 20 with RA) treated with anti-TNF therapy and a group of 8 subjects with rheumatoid diseases (including 8 with RA) receiving standard treatment. The above-mentioned study documented, after anti-TNF treatment, a significant reversal of the initial subclinical left ventricular systolic dysfunction manifested by improvement in the peak systolic circumferential strain and peak diastolic circumferential strain rate [20]. Similar conclusions may be drawn on the basis of studies by Kobayashi et al. and Lehmonen et al. [21, 22]. A study by Kobayashi et al. analysed the effect of tocilizumab treatment on the left ventricular systolic function. At baseline, a group of 13 subjects with RA was characterised by lower-peak systolic regional radial strain than a group of 10 healthy subjects. Fifty-two weeks later, subjects with RA treated with tocilizumab were characterised by higher-peak systolic regional radial strain than at baseline [21]. On the other hand, a study by Lehmonen et al. analysed the effect of active RA treatment (regardless of the adopted treatment regimen) on the dynamics of heart changes assessed by CMR. A year-long treatment of active RA, in a group of 39 subjects, resulted in the improvement of peak diastolic mean mid-short-axis circumferential strain rate of all six segments, but the highest improvement was observed for the anterior segment [22].

Results of the analysed studies are basically consistent with the cited data from studies of other research teams. It must be stated, however, that the majority of the above-mentioned studies, as well as our study, demonstrated deterioration in the left ventricular systolic function in subjects with RA assessed by strain, with no such deterioration using classic assessment by left ventricular ejection fraction. This proves that left ventricular myocardial strain may be “added value" to the ejection fraction (typically used in assessing left ventricular systolic function), which provides higher sensitivity for lesion detection, especially in asymptomatic patients. It must also be emphasised that only in studies by Lehmonen et al. [22], as well as in the present study, the attempt has been made to conduct segmental analysis of left ventricular myocardial strain; on the other hand, only studies by Ntusi et al. [5] showed the significance of global strain values as an indicator of deteriorated left ventricular systolic function in RA patients, which was also pointed out in our study.

In the studies conducted so far, left ventricular myocardial strain in subjects with rheumatoid arthritis was much more frequently analysed by echocardiography. Over the last few years, such studies have been published by, for example, Naseem et al., Baktir et al. and Fine et al., indicating a deterioration of thus-assessed left ventricular systolic function in subjects with RA versus healthy subjects [23–25]. The achieved results confirm the data from the studies using echocardiography. According to the authors, this is a valuable indirect argument against the criticism of echocardiographic assessment of the left ventricular strain. However, it should be remembered, at the same time, the superiority of CMR over echocardiography. Using CMR, in contrast to echocardiography, it is possible to additionally assess the morphology of the myocardium, using the standard sequences: LGE, STIR, T1-, T2-mapping or ECV.

The achieved results also make it possible to attempt the determination of the usefulness of selected clinical and laboratory parameters characterising the severity and course of RA for the purposes of risk prediction with regard to reduced left ventricular systolic function assessed by CMR strain in the study group of patients. The regression analysis showed that for the analysed clinical and laboratory variables characterising the severity and course of RA, lower-peak GLS was associated with higher activity of antibodies against cyclic citrullinated peptide and higher concentration of neutrophil gelatinase-associated lipocalin, while lower-peak GCS was associated with higher activity of antibodies against cyclic citrullinated peptide. The achieved results may indicate higher anti-CCP activity as the most useful laboratory predictor of deteriorated left ventricular systolic function in the study group of patients.

Studies on the relationship between NGAL level and changes in the heart morphology and function assessed by cardiac magnetic resonance have not been published, so far. A demonstration in our study relationship, independent of other variables, between a higher NGAL level and lower-peak GLS, may be considered a novel aspect of the conducted studies. A relationship between the anti-CCP activity and changes in the cardiac magnetic resonance was shown by studies conducted by Giles et al. The study revealed that higher anti-CCP was correlated with lower left ventricular mass, lower end-diastolic volume and lower left ventricular ejection fraction; at the same time, no relationship was revealed between anti-CCP and left ventricular ejection fraction [8]. However, the relationships between anti-CCP and peak GLS, and between anti-CCP and peak GCS revealed in the present study may indicate the significance of anti-CCP activity for the left ventricular systolic function.

The present study has some limitations. First of all, the lack of CMR sequences to assess the morphology of the left ventricular myocardium (LGE, T1-mapping, T2-mapping, ECV) must be mentioned. One of the basic limitations of this study is also a small number of the study subjects. One may also discuss the purposefulness of excluding patients with clinically manifested heart damage and at the same time including patients with certain risk factors for the cardiovascular system, i.e. diabetes, arterial hypertension or smoking cigarettes. Another limitation is a subjective selection of laboratory parameters characterising the severity and course of RA, and of disease activity scales. An important shortcoming is also disregarding the parameters of strain assess by a different technique, e.g. echocardiography.

## Conclusions

1. In subjects with no clinically manifested cardiac damage, rheumatoid arthritis is associated with a deteriorated left ventricular systolic function assessed by left ventricular myocardial strain measured by cardiac magnetic resonance feature tracking.
2. Deteriorated left ventricular systolic function in RA patients refers to both global and regional strain parameters. Lower global contractility in RA patients refers to longitudinal and circumferential shortening. Lower regional contractility refers to specific segments, especially associated with the interventricular septum and left ventricular lateral wall.
3. The occurrence of rheumatoid arthritis is, aside to the occurrence of arterial hypertension, to increase the activity of antibodies against cyclic citrullinated peptide and higher concentration of neutrophil gelatinase-associated lipocalin and independent risk factor for reduced peak global longitudinal strain.
4. The occurrence of rheumatoid arthritis is, similarly to the occurrence of diabetes, tobacco smoking, higher activity of antibodies against cyclic citrullinated peptide and current use of methotrexate, and independent risk factor for reduced peak global circumferential strain. And the current use of steroids in the therapy constitutes an independent protecting factor against reduced peak global circumferential strain.

## Data Availability

Study data can be made available upon documented request.

## Abbreviations

aIVSDD: Anterior interventricular septum diastolic dimension
anti-CCP: Antibodies against cyclic citrullinated peptide
Ao: Aortic root
BMI: Body mass index
BSA: Body surface area
CMR: Cardiac magnetic resonance
CRP: C-reactive protein
CS: Circumferential strain
CVD: Cardiovascular disease
EDV: End diastolic volume
EF: Ejection fraction
ESV: End-systolic volume
GCS: Global circumferential strain
GLS: Global longitudinal strain
GRS: Global radial strain
LA: Left atrium
LS: Longitudinal strain
LV: Left ventricular
LVEDD: Left ventricular end diastolic dimension
LVESD: Left ventricular end-systolic dimension
LVM: Left ventricular mass
LVMI: Left ventricular mass index
NGAL: Neutrophil gelatinase-associated lipocalin
PWDD: Posterior wall diastolic dimension
RA: Rheumatoid arthritis
RF: Rheumatoid factor
RIQAS: Randox International Quality Assessment Scheme
RS: Radial strain
SV: Stroke volume
